# Early infant feeding practices in three African countries: the PROMISE-EBF trial promoting exclusive breastfeeding by peer counsellors

**DOI:** 10.1186/1746-4358-9-19

**Published:** 2014-11-18

**Authors:** Ingunn Marie S Engebretsen, Victoria Nankabirwa, Tanya Doherty, Abdoulaye Hama Diallo, Jolly Nankunda, Lars Thore Fadnes, Eva-Charlotte Ekström, Vundli Ramokolo, Nicolas Meda, Halvor Sommerfelt, Debra Jackson, Thorkild Tylleskär, James K Tumwine, For the PROMISE-EBF study group

**Affiliations:** Centre for International Health, Department of Global Public Health and Primary Health Care, University of Bergen, Bergen, Norway; School of Public Health, College of Health Sciences, Makerere University, Kampala, Uganda; Health Systems Research Unit, Medical Research Council, Cape Town, South Africa; School of Public Health, University of the Western Cape, Cape Town, South Africa; Centre MURAZ, Ministry of Health, Bobo-Dioulasso, Burkina Faso; Department of Paediatrics and Child Health, College of Health Sciences, Makerere University, Kampala, Uganda; Department of Clinical Dentistry, University of Bergen, Bergen, Norway; Department Women’s and Children’s Health, Uppsala University, Uppsala, Sweden; UNICEF, New York, USA; Department of Global Public Health, Norwegian Institute of Public Health, Kragujevac, Norway

**Keywords:** Trial, Exclusive breastfeeding, Peer-counselling, Colostrum, Prelacteal feeding

## Abstract

**Background:**

Immediate and exclusive initiation of breastfeeding after delivery has been associated with better neonatal survival and child health and are recommended by the WHO. We report its impact on early infant feeding practices from the PROMISE-EBF trial.

**Methods:**

PROMISE-EBF was a cluster randomised behaviour change intervention trial of exclusive breastfeeding (EBF) promotion by peer counsellors in Burkina Faso, Uganda and South Africa implemented during 2006-2008 among 2579 mother-infant pairs. Counselling started in the last pregnancy trimester and mothers were offered at least five postnatal visits. Early infant feeding practices: use of prelacteal feeds (any foods or drinks other than breast milk given within the first 3 days), expressing and discarding colostrum, and timing of initiation of breastfeeding are presented by trial arm in each country. Prevalence ratios (PR) with 95% confidence intervals (95%CI) are given.

**Results:**

The proportion of women who gave prelacteal feeds in the intervention and control arms were, respectively: 11% and 36%, PR 0.3 (95% CI 0.2, 0.6) in Burkina Faso, 13% and 44%, PR 0.3 (95% CI 0.2, 0.5) in Uganda and 30% and 33%, PR 0.9 (95% CI 0.6, 1.3) in South Africa. While the majority gave colostrum, the proportion of those who expressed and discarded it in the intervention and control arms were: 8% and 12%, PR 0.7 (95% CI 0.3, 1.6) in Burkina Faso, 3% and 10%, PR 0.3 (95% CI 0.1, 0.6) in Uganda and 17% and 16%, PR 1.1 (95% CI 0.6, 2.1) in South Africa. Only a minority in Burkina Faso (<4%) and roughly half in South Africa initiated breastfeeding within the first hour with no large or statistically significant differences between the trial arms, whilst in Uganda the proportion of early initiation of breastfeeding in the intervention and control arms were: 55% and 41%, PR 0.8 (95% CI 0.7, 0.9).

**Conclusions:**

The PROMISE-EBF trial showed that the intervention led to less prelacteal feeding in Burkina Faso and Uganda. More children received colostrum and started breastfeeding early in the intervention arm in Uganda. Late breastfeeding initiation continues to be a challenge. No clear behaviour change was seen in South Africa.

**Trial registration:**

NCT00397150.

**Electronic supplementary material:**

The online version of this article (doi:10.1186/1746-4358-9-19) contains supplementary material, which is available to authorized users.

## Background

Despite substantial reduction in child mortality, an almost stagnant risk of death within the first weeks of life is a reality in sub-Saharan Africa [1]. Recent reports found an annual rate of reduction in neonatal mortality from 40.5/1000 in 2000 to 35/1000 in 2010 [1]. Efforts have been made to identify interventions that are safe, affordable, acceptable and can be scaled up in order to effectively reduce early child morbidity and mortality [2]. Exclusive breastfeeding has been identified as one such intervention. In 2006 delayed and non-exclusive breastfeeding were described as major risk factors for neonatal deaths in a large cohort from a vitamin A trial in Ghana (ObaapaVitA trial) [3]. Furthermore, there was an increased risk of dying from infectious diseases in partially breastfed and even in predominantly breastfed newborns compared to those that only received breast milk [4]. Early and exclusive breastfeeding is recommended by stakeholders [5] and is described as the best option with respect to immunological and nutritional values [6]. The three public health early infant feeding pillars include a) initiating breastfeeding as early as possible, and not later than 1 hour after birth, b) exclusive breastfeeding that includes avoiding pre-lacteal feeds, and c) giving the colostrum to the child [5].

In many sub-Saharan settings early infant feeding practices have been influenced by a variety of less favourable habits, both cultural and propagated by health facilities, such as separation from the mother, early cord clamping, early bathing of baby and separate cleansing rituals of the mother before initiation of breastfeeding [7], and routine feeding. These habits have been practiced rather than the recommended early skin-to-skin contact, immediate breastfeeding and feeding-on-demand [7, 8]. Furthermore, traditional practices such as expressing and discarding of colostrum due to a belief it contains dirt, giving feeds as part of religious ceremonies and other rituals have also disturbed the vulnerable early feeding period [9, 10].

The Lancet Nutrition Interventions Review Group recently presented a summary of existing evidence for improving maternal and child nutrition [11] where early initiation of exclusive breastfeeding was one of the key interventions discussed. Understanding community behaviour change interventions is important in order to build more integrated and comprehensive contextualised programmes in the future. In the PROMISE-EBF trial [12] pregnant women in the intervention group received counselling by peers supporting and promoting exclusive breastfeeding from late pregnancy throughout the first half year of their babies’ lives. The control group had no peer-counselling intervention. We present findings of early infant feeding practices including: a) initiation of breastfeeding; b) use of prelacteal feeds, and c) whether colostrum was discarded.

## Methods

The design and undertaking of the trial has been described previously [12]. Briefly, the study was conducted in three countries: 1) Banfora in south-west Burkina Faso, a rural area dominated by subsistence farming; 2) in eastern Uganda, the sites comprised rural Bungokho where both subsistence farming and petty trading are common, and urban Mbale Municipality characterised by informal settlements and small industries; and 3) in South Africa, three geographically separate sites included Paarl, a commercial farming area in the Western Cape Province, peri-urban Umlazi, and the rural Rietvlei in KwaZulu-Natal. Infant mortality rates (IMRs) at the time of the study were 92/1,000 in Burkina Faso and 85/1,000 in Uganda. In South Africa the IMRs were 40/1,000 in Paarl, 60/1,000 in Umlazi and 99/1,000 in Rietvlei [13]. Furthermore, at the time of the study HIV-prevalence and also access to testing and prevention-of-mother-to-child transmission of HIV (PMTCT) services varied considerably between the three countries: In Burkina Faso and Uganda the estimated prevalence of maternal HIV was 6% while it was 29% in South Africa [14, 15].

Cluster-randomisation was 1:1, stratified by country and sites. In Burkina Faso, randomisation was not stratified as the clusters were considered homogeneous at baseline with regard to sociodemographic characteristics; in Uganda, it was stratified according to urban and rural location, and in South Africa according to the three sites which were geographically different. A cluster comprised 1-2 villages or communities with an average of 1000 inhabitants (~35 infants born per year, i.e. a birth rate of 3.5%). Clusters were selected in close collaboration with community leaders. Care was taken to allow for ‘corridors’ between selected clusters to avoid potential contamination across clusters. In each site, clusters were randomised to either the intervention (EBF peer-counselling) or the control arm, where EBF was not promoted by our research team. In South Africa, peer support for families to obtain birth certificates and social welfare grants by separate counsellors was provided in the control clusters.

The peer counsellors supporting and promoting EBF for six months were from the same communities as the mothers. They were trained in a one week course by the national research teams with a curriculum from the WHO courses ‘*Breastfeeding Counselling: a Training Course*’ [16] and ‘*HIV & Infant Feeding Counselling: a Training Course*’ [17] adapted for the sites. This curriculum came with basic illustrations that were used to ease discussion of latching techniques and positioning of the baby and mother. The curriculum was thoroughly pretested [18] and the research team addressed in detail the pre- and post- training knowledge among the peer counsellors [19]. The WHO curriculum was supplemented with basic training because it focuses only on breastfeeding excluding several aspects such as the need for community acceptance and permission from the husbands. To meet these needs, the curriculum was adapted to the local context, particularly paying attention to commonly used traditional and cultural liquids given to newborns. The peer counsellors’ knowledge was assessed and found satisfactory before implementation and they were followed up at least monthly during the intervention period. The sessions with the mother included theoretical knowledge on exclusive breastfeeding and the peers also got knowledge to guide mothers having common difficulties like engorgement and sore nipples; and to recognise more serious problems like mastitis and abscesses. The sessions with the mothers were mostly interactive processes and were scheduled to fit the mother’s programme in her home. All mothers were offered at least five visits, one of which occurred towards the end of pregnancy. Roughly speaking they lasted from 15 minutes to an hour, but those data are not valid for quantitative analysis. The intervention is described previously [12, 19–21].

Pregnant women residing in the clusters, intending to breastfeed and with no intention to move were recruited into the study. HIV status was not a recruitment consideration. In Uganda eligible women were identified by recruiters in the study areas and thereafter approached for data collection. In Burkina Faso and South Africa, a random sample of mothers in the intervention clusters were approached for data collection as the number of women receiving the intervention in the clusters exceeded the sample size requirements. Exclusion criteria for data collection were severe psychological illness which could interfere with consent or trial collaboration, and having given birth more than 1 week before the first data collection contact. The mother-infant pairs were assessed three weeks after delivery and if the child had any severe malformation that could interfere with breastfeeding they were excluded from data collection.

Sample size calculation was done for EBF and diarrhoea prevalence at 12 weeks [12], not for the primary outcome of this paper. A full trial profile by arm and country is already published [12, 22]. The analysis included 2,579 singleton live children. A post hoc power calculation found very high power (0.8-1) for the detected differences in prelacteal feeding, early initiation of breastfeeding and not giving colostrum (Additional file 1). Data collection was done from 2006 to 2008 by trained interviewers in a series of five interviews: a recruitment visit late in pregnancy, and visits at 3 (timely visit range: 1.5–4.5) weeks; 6 (4.5–9) weeks; 12 (9–18) weeks; and 24 (18–28) weeks after birth. The first interview focused mainly on sociodemographic and socioeconomic characteristics. The follow-up visits addressed feeding patterns, infant illness, and anthropometric measurements. Early infant feeding practices were assessed in the first interview after birth. If the respondent was not available at that interview, the next two interviews provided an opportunity to answer the early infant feeding questions.

### Definitions

Structured questionnaires were developed and adapted from the literature [23, 24] as well as from prior work in the participating countries [25–27]. Prelacteal feeding was defined as any non-breast milk feeds given within the first three days after birth. The women were asked if the baby was given 1) any mouthfuls to drink other than breast milk within the first three days after birth, and thereafter 2) any drops of something to taste. The latter was intended to capture religious or traditional practices. If they answered ‘yes’ to prelacteal feeds they were asked to answer yes or no to a predefined food list of 16 items that were identified through formative research [25]. Regarding giving or not giving colostrum the women were asked if they gave the first milk to the baby or expressed and discarded it. Initiation of breastfeeding was determined by the timing of the first breastfeed. The following cumulative categories were created based on categorical data collection: Within the first hour; within 12 hours; within 24 hours and after.

### Analysis

Factors associated with the non-recommended early feeding practices prelacteal feeds and late (after 12 hours) initiation of breastfeeding were studied. The factors of interest related to the mother were: *maternal body mass index*, kg/m^2^ (BMI) at six weeks; *place of delivery,* ‘facility delivery’ contrasting ‘home delivery’*; type of delivery,* ‘normal vaginal’ contrasting ‘Caesarean-section, breech or other complications’; *parity; socioeconomy* where the two top quintiles contrasted the 3 bottom quintiles, the quintiles were derived from multiple correspondence analysis [12, 28]; *education* given as years of schooling; *antenatal care* attendance; and the *intervention* package. Factors related to the child were *body size* and *gender.* The PROMISE-EBF trial did not capture birth weight, however, we asked for birth weight at the 3-week interview. In Burkina Faso, 98% could not report any birth weight, contrasting 65% in Uganda and 8% in South Africa. We therefore had limited and unreliable birth-weight data. As a proxy for size at birth, we used the 3-week anthropometric data, weight-for-length, length-for-age and weigh-for-age z-scores according to the WHO Child Growth standards [29], acknowledging the limitation that early infant feeding practices may have influenced the anthropometric status of the child at 3 weeks.

Descriptive statistics included means with 95% confidence intervals (CI), medians and ranges for continuous variables and proportions for categorical variables. For the categorical data, generalised linear models of the binomial family with a log link were used to calculate prevalence ratios (PR). This was done a) by trial allocation and b) according to a cohort design where statistically significant factors associated with the outcome at the p <0.05 level, socio-economic and the trial allocation went into the multi-variable models. Analysis was done by country and all presented data were adjusted for the design effect by having randomized *clusters* rather than individuals. The data were analysed using STATA 11 SE (Stata Corp LP, College Station, TX, USA).

### Ethical approval

Approval for the trial (ClinicalTrials.gov: NCT00397150) was obtained from the following bodies: 1) Burkina Faso: Institutional Review board of Centre Muraz (No 013/2005/CE-CM) and the Ministry of Health at national and regional level; 2) Uganda: Makerere University Faculty of Medicine Research and Ethics Committee, and the Uganda National Council for Science and Technology; 3) South Africa: Ethics Committee of the Medical Research Council South Africa; and 4) Norway: Regional Committees for Medical and Health Research Ethics (REK VEST), 8 Sept 2005, issue number 05/8197. Women provided verbal informed consent for participation in the peer-counselling programme, which was regarded as a service. Written informed consent for participation in the study was signed or thumb-printed by each respondent.

## Results

### Population

2579 mother-infant pairs were enrolled in the study in the three countries: n =794 in Burkina Faso, n =765 in Uganda and n =1020 in South Africa. Two publications [12, 22] provide exact details on baseline characteristics in the trial by country and trial allocation, indicating a fairly successful randomisation and highlighting huge country variations particularly in socioeconomic and education levels where Burkina Faso is the poorest and South Africa the least poor among the three. The majority were seen at the 3-week visit: 729/794 (91.8%) in Burkina Faso, 731/765 (95.6%) in Uganda and 951/1020 (93.2%) in South Africa for the early infant feeding recall. A small proportion was interviewed for the early infant feeding recall at 6 weeks and at 12 weeks, respectively: Burkina Faso: 47/794 (5.9%), Uganda: 25/765 (3.3%), South Africa: 49/1020 (4.8%) and Burkina Faso: 18/794 (2.3%), Uganda: 3/765 (0.4%), South Africa: 14/1020 (1.4%). The median, days, (percentile, p, p25-p75) recall time for prelacteal feeds were 22 (19-25) in Burkina Faso, 22 (21-24) in Uganda and 24 (21-30) in South Africa.

### Prelacteal feeds and drops or religious feeds

The prevalence of giving prelacteal feeds in the intervention and control groups were: 11% and 37%, PR 0.3 (95% CI 0.2, 0.6) in Burkina Faso, 13% and 43%, PR 0.3 (95% CI 0.2, 0.5) in Uganda and 30% and 33%, PR 0.9 (95% CI 0.6, 1.3) in South Africa (Table 1). A similar prevalence ratio was seen in Burkina Faso and Uganda for the habit of giving drops or religious feeds: PR 0.4 (95% CI 0.2, 0.8) and PR 0.3 (95% CI 0.3, 0.4), but this was not seen in South Africa. The groups reporting they were giving either prelacteal feeds, drops or religious feeds were to a large extent overlapping (lowest degree of overlap in a study group: 62%). Types of prelacteal feeds given are presented in Table 2.

**Table 1:** **Early infant feeding practices by study arm in the three countries**

	Intervention	Control	PR (95% CI)
**Burkina Faso**			
Prelacteal feeds	41/372 (11.0)	135/371 (36.4)	0.30 (0.17,0.55)
Religious/ritual drops	43/371 (11.6)	108/371 (29.1)	0.40 (0.19,0.82)
Feeds/drops combined	57/372 (15.3)	160/371 (43.1)	0.36 (0.18,0.69)
Initiation (cum):^1^			
Within 1 h	14/392 (3.6)	14/402 (3.5)	0.99 (0.96,1.04)
Within 12 h	207/392 (52.8)	179/402 (44.5)	0.85 (0.65,1.12)
Within 24 h	322/392 (82.1)	298/402 (74.1)	0.69 (0.46,1.04)
No colostrum	30/371 (8.1)	46/371 (12.4)	0.65 (0.26,1.63)
**Uganda**			
Prelacteal feeds	52/387 (13.4)	153/352 (43.5)	0.31 (0.21,0.45)
Religious/ritual drops	56/387 (14.5)	151/352 (42.9)	0.34 (0.27,0.42)
Feeds/drops combined	73/387 (18.9)	194/352 (55.1)	0.34 (0.26,0.46)
Initiation (cum):^1^			
Within 1 h	219/396 (55.3)	151/369 (40.9)	0.76 (0.68,0.85)
Within 12 h	360/396 (90.9)	297/369 (80.5)	0.47 (0.34,0.63)
Within 24 h	376/396 (95.0)	335/369 (90.8)	0.55 (0.31,0.95)
No colostrum	10/382 (2.6)	33/349 (9.5)	0.28 (0.13,0.61)
**South Africa**			
Prelacteal feeds	145/480 (30.2)	144/437 (33.0)	0.92 (0.64,1.32)
Religious/ritual drops	55/220 (25.0)	43/198 (21.7)	1.15 (0.74,1.78)
Feeds/drops combined	148/482 (30.7)	145/440 (33.0)	0.93 (0.64,1.34)
Initiation (cum):^1^			
Within 1 h	252/535 (47.1)	248/485 (51.1)	1.08 (0.72,1.62)
Within 12 h	415/535 (77.6)	376/485 (77.5)	0.99 (0.65,1.53)
Within 24 h	454/535 (84.9)	413/485 (85.2)	1.02 (0.63,1.64)
No colostrum	83/478 (17.4)	68/439 (15.5)	1.12 (0.60,2.11)

^1^Categories are cumulative.

The table includes: Prelacteal feeds, religious/ritual drops, and prelacteal feeds and drops combined the first 3 days after birth; initiation of breastfeeding within 1, 12 and 24 hours (h) after birth; and, expressing/discarding colostrum (no colostrum).

**Table 2:** **Types of prelacteal feeds given, n (%)**

Feed ^1^	Burkina Faso		Uganda		South Africa	
	Intervention	Control	Intervention	Control	Intervention	Control
**Water**	14 (3.6)	88 (21.9)	21 (5.3)	36 (9.8)	31 (5.8)	29 (6.0)
**Sugar water**	6 (1.5)	9 (2.2)	30 (7.6)	117 (31.7)	45 (8.4)	51 (10.5)
**Salt water**	0	0	5 (1.3)	25 (6.8)	2 (0.4)	3 (0.6)
**Infant formula**	0	0	1 (0.3)	0	36 (6.7)	34 (7.0)
**Traditional medicine**	16 (4.1)	34 (8.5)	1 (0.3)	0	28 (5.2)	22 (4.5)
**Other** ^**2**^	6 (1.5)	28 (7.0)	1 (0.3)	1 (0.3)	5 (0.9)	6 (1.2)

^1^These items were given to 1% or less in all three countries: Diluted cow’s milk, undiluted cow’s milk, any other powdered milk, porridge, soup, honey.

^2^Other: Mothers in Burkina Faso gave mostly a variety of fruit juices, orange and lemon drops. Mothers in South Africa mostly gave a variety of gripe water/purchased drinks and malt.

### Colostrum

The majority in all three countries gave colostrum to their babies (Table 1). There were no substantial or statistical significant differences between women in the intervention and control groups regarding this behaviour in Burkina Faso or South Africa, but in Uganda, those in the intervention group discarded colostrum less frequently (3%) than those in the control groups (10%), PR 0.3 (95% CI 0.1, 0.6).

### Initiation of breastfeeding

Only a minority in Burkina Faso (<4%) and roughly half in South Africa initiated breastfeeding within the first hour with no substantial or statistically significant differences between the intervention and control groups. In Uganda the women initiated breastfeeding early to a slightly larger extent than in the other two countries, and women in the intervention group even more so than in the control group (Table 1).

### Factors influencing early infant feeding practices

Factors related to prelacteal feeding (Tables 3 and 4) and late (>12 hours) initiation of breastfeeding (Tables 5 and 6) were studied separately. The following factors were found to be associated in the adjusted analysis with prelacteal feeds: In Burkina Faso being among the least poor 2/5 in the socioeconomic ranking, having antenatal care visits, getting the intervention, as well as relatively high WLZ were associated with not giving prelacteal feeds. In South Africa this also applied for WLZ. In Uganda antenatal care visits were borderline statistically associated with the prevalence of prelacteal feeding, thus, an opposite result from Burkina Faso. The trial was associated with less prelacteal feeding.

**Table 3:** **Prelacteal feeding by sociodemographic characteristics, continuous data presented**

Continuous variables ^1^	Burkina Faso	Uganda	South Africa
**Mother BMI** ^**2**^ **, mean, (95%CI)**	1.04 (0.96, 1.12)	1.03 (0.99,1.07)	1.01 (0.99, 1.03)
**Child WLZ** ^**3**^ **, mean (95% CI)**	0.86 (0.75, 0.98)	1.10 (0.99,1.21)	0.89 (0.80, 0.98)
**Child LAZ** ^**4**^ **, mean (95% CI)**	1.10 (0.99, 1.23)	1.07 (0.95,1.20)	1.03 (0.94, 1.13)
**Maternal education, mean years school, (95% CI)**	1.04 (0.98, 1.11)	1.01 (0.98,1.05)	0.97 (0.93, 1.02)

^1^Crude bivariable analysis remained unchanged in adjusted analysis.

^2^BMI, Body-mass-index (kg/m2).

^3^WLZ, Weight-for-length z-scores.

^4^LAZ, Length-for-age z-scores.

**Table 4:** **Prelacteal feeding by sociodemographic characteristics, categorical data presented**

Categorical variables ^1^	Burkina Faso			Uganda			South Africa		
	Prelacteals(n)	c PR	a PR	Prelacteals(n)	c PR	a PR	Prelacteals(n)	c PR	a PR
**Antenatal care**	n/N (%)			n/N (%)			n/N (%)		
No	65/200 (32.5)	1	1	35/176 (19.9)	1	1	7/14 (50.0)	1	1
Yes	111/539 (20.6)	0.63 (0.45, 0.89)	0.73 (0.54, 0.98)	159/527 (30.2)	1.52 (1.08, 2.14)	1.44 (0.99, 2.09)	279/898 (31.1)	0.62 (0.37, 1.04)	0.64 (0.40, 1.03)
**Facility delivery**									
No	98/418 (23.4)	1		92/324 (28.4)	1		31/49 (63.3)	1	
Yes	77/324 (23.8)	1.01 (0.68, 1.51)		110/408 (27.0)	0.95 (0.75, 1.20)		255/863 (29.6)	0.45 (0.36, 0.61)	
**C, section/complicated delivery**									
No	175/735 (23.8)	1		192/709 (27.1)	1	1	234/718 (32.6)	1	1
Yes	1/8 (12.5)	0.53 (0.07, 3.73)		13/30 (43.3)	1.60 (1.02, 2.52)	1.42 (0.94, 2.14)	55/199 (27.6)	0.85 (0.63, 1.14)	0.75 (0.53, 1.07)
**Parity**									
Primipara	31/121 (25.6)	1		47/159 (29.6)	1		135/457 (29.5)	1	
Mulitipara	145/621 (23.4)	0.91 (0.60, 1.38)		154/573 (26.9)	0.91 (0.68, 1.22)		154/460 (33.5)	1.13 (0.94, 1.36)	
**Sex of child, girl**									
Boy	97/386 (25.1)	1		102/375 (27.2)	1		144/450 (32.0)	1	
Girl	79/356 (22.2)	0.88 (0.71, 1.10)		103/364 (28.3)	1.04 (0.81, 1.34)		145/465 (31.2)	0.97 (0.80, 1.19)	
**Top 40% on wealth index**									
No	125/448 (27.9)	1	1	124/444 (27.3)	1	1	180/531 (33.9)	1	1
Yes	51/294 (17.4)	0.62 (0.39, 0.98)	0.64 (0.43, 0.96)	81/295 (27.5)	0.98 (0.79, 1.22)	0.85 (0.69, 1.03)	109/386 (28.2)	0.83 (0.67, 1.04)	0.89 (0.69, 1.14)
**Trial arm allocation**									
Control	135/371 (36.4)	1	1	153/352 (43.4)	1	1	144/437 (33.0)	1	1
Intervention	41/372 (11.0)	0.30 (0.17, 0.56)	0.29 (0.18, 0.49)	52/387 (13.4)	0.31 (0.21, 0.44)	0.32 (0.21, 0.47)	145/480 (30.2)	0.92 (0.64, 1.31)	0.82 (0.55, 1.21)

^1^The following where included in the final model: Burkina Faso: Intervention, socio,economic, antenatal care and WLZ; Uganda: Intervention, delivery, socio,economic and antenatal care; and South Africa: Intervention, delivery, socio,economic demographics, antenatal care and WLZ.

cPR: crude Prevalence Ratios, aPR: adjusted Prevalence Ratios.

**Table 5:** **Late initiation of breastfeeding (after 12 hours) by sociodemographic characteristics, continuous data presented**

Continuous variables ^1^	Burkina Faso	Uganda	South Africa
**Mother BMI** ^**2**^ **, mean,95%CI**	1.01 (0.98, 1.05)	0.94 (0.88, 1.02)	1.04 (1.04, 1.05)
**Child WLZ** ^**3**^ **, mean 95% CI** ^**2**^	0.99 (0.92, 1.07)	0.90 (0.75, 1.08)	1.02 (0.89, 1.16)
**Child LAZ** ^**4**^ **, mean 95% CI** ^**2**^	0.93 (0.89, 0.98)	0.99 (0.79, 1.26)	0.93 (0.83, 1.05)
**Mother, mean years school, 95% CI**	0.98 (0.94, 1.02)	0.96 (0.92, 1.00)	1.02 (0.94, 1.09)

^1^Crude bivariable analysis remained unchanged in adjusted analysis.

^2^BMI, Body-mass-index (kg/m2).

^3^WLZ, Weight-for-length z-scores.

^4^LAZ, Length-for-age z-scores.

**Table 6:** **Late initiation of breastfeeding (after 12 hours) by sociodemographic characteristics, categorical data presented**

Categorical variables ^1^	Burkina Faso			Uganda			South Africa		
	Late BF	c PR	a PR	Late BF	c PR	a PR	Late BF	c PR	a PR
**Antenatal care**	n/N (%)			n/N (%)			n/N (%)		
No	130/221 (58.8)	1	1	26/182 (14.3)	1		5/16 (31.3)	1	
Yes	277/569 (48.7)	0.83 (0.68, 1.01)	0.89 (0.71, 1.11)	74/546 (13.6)	0.95 (0.59, 1.53)		222/997 (22.3)	0.71 (0.30, 1.72)	
**Facility delivery**									
No	226/418 (54.1)	1	1	35/324 (10.8)	1		16/52 (30.8)	1	1
Yes	130/324 (40.1)	0.74 (0.59, 0.93)	0.76 (0.61, 0.95)	43/408 (10.5)	0.97 (0.66, 1.44)		172/923 (18.6)	0.61 (0.38, 0.97)	0.43 (0.27, 0.68)
**C. section/complicated delivery**									
No	353/735 (48.0)	1		66/709 (9.3)	1	1	116/753 (15.4)	1	1
Yes	55/59 (93.2)	1.94 (1.61, 2.34)		42/56 (75.0)	8.06 (5.86, 11.08)	6.90 (5.17, 9.22)	113/267 (42.3)	2.75 (2.08, 3.62)	2.29 (1.70, 3.10)
**Parity**									
Primipara	68/129 (52.7)	1		29/166 (17.5)	1		127/508 (25.0)	1	1
Multipara	340/664 (51.2)	0.97 (0.80, 1.18)		77/592 (13.0)	0.74 (0.53, 1.04)		102/512 (19.9)	0.80 (0.64, 0.99)	0.71 (0.54, 0.93)
**Sex of child, girl**									
Boy	214/406 (52.7)	1		55/387 (14.2)	1		102/498 (20.5)	1	
Girl	194/387 (50.1)	0.95 (0.83, 1.10)		50/375 (13.3)	0.94 (0.59, 1.49)		118/512 (23.1)	1.13 (0.88, 1.45)	
**Top 40% on wealth index**									
No	238/478 (49.8)	1	1	76/461 (16.5)	1	1	134/586 (22.9)	1	1
Yes	170/315 (54.0)	1.08 (0.96, 1.23)	1.06 (0.93, 1.23)	32/304 (10.5)	0.64 (0.42, 0.97)	0.78 (0.62, 0.99)	95/434 (21.9)	0.96 (0.70, 1.31)	0.92 (0.67, 1.26)
**Trial arm allocation**									
Control	223/402 (55.5)	1	1	72/369 (19.5)	1	1	109/485 (22.5)	1	1
Intervention	185/392 (47.2)	0.85 (0.65, 1.12)	1.01 (0.68, 1.51)	36/396 (9.1)	0.47 (0.34, 0.63)	0.59 (0.46, 0.76)	120/535 (22.4)	1.00 (0.65, 1.53)	1.01 (0.69, 1.50)

^1^The following went into the adjusted model: Burkina Faso: Intervention, birthplace, socio, economic and antenatal care; Uganda Intervention, delivery and socio,economic; and South Africa: Intervention, birthplace, delivery, parity and socio, economic demographics.

cPR: crude Prevalence Ratios, aPR: adjusted Prevalence Ratios.

Caesarean section or complicated delivery was a risk factor for late initiation of breastfeeding in all three countries. Further, delivery in a facility was associated as a protective factor in Burkina Faso and South Africa. Being multipara was only associated with early initiation of breastfeeding in South Africa, as well as receipt of the intervention and higher socioeconomic ranking in Uganda.

## Discussion

This study assessed early infant feeding practices including prelacteal feeding within the first three days, expressing and discarding of colostrum and initiation of breastfeeding. The study investigated how a peer counselling intervention was related to these early feeding practices in three African countries. Further, it has also examined how other exposures might have influenced early infant feeding.

The intervention resulted in less prelacteal feeding in Burkina Faso and Uganda. More children received colostrum in Uganda, while no clear behaviour change was seen in South Africa. Initiation of breastfeeding was predominantly much later than recommended. The intervention itself was a major contributor to changes in early infant feeding practices, however other factors mattered in the different countries, such as socioeconomic factors, antenatal care visits, place of delivery and size of the baby. Caesarean section or complicated delivery was associated with more prelacteal feeding and delayed breastfeeding which has been described in the literature [30]. These findings are in accordance with emphasizing biological, societal and educational factors as important for early infant feeding practices [30], thus strengthening the arguments for comprehensive support for early child feeding. In our trial antenatal care and facility delivery were associated with early feeding practices, but not in a consistent manner. Thus, our research group suggests system strengthening and more support supervision to health workers that assist mothers during pregnancy, delivery and early child care.

Our finding of early feeding behaviour change in Burkina Faso and Uganda, but not in South Africa is in line with what we described in the prior publication on exclusive breastfeeding practices at three months of infant age [12]. Generally, the intervention was associated with almost a doubling of EBF prevalence in all three countries, but with the greatest changes in absolute prevalence in Uganda and Burkina Faso from around forty to eighty percent at 12 weeks.

### Differences and challenges in the peer counselling intervention in the three different countries

A post-intervention study in Uganda described that the mothers expressed satisfaction with the intervention [31] and a tendency towards the peer counsellor evolving from teacher to friend during the study period. The mothers expressed satisfaction with the taught content and expressed a desire to continue themselves as peer counsellors. The mothers reported an appreciation of culturally-sensitive behaviour change messages. One limitation with our knowledge about this intervention in Uganda was the researcher’s involvement in the intervention implementation and lack of external evaluation [19, 31]. This could in a way have led to under reporting of problems; however, this role was carefully reflected upon [32]. A described strength of the implementation in Uganda and Burkina Faso was that the teams involved the communities to a high degree, also in the selection of peers [19, 33].

In South Africa cultural issues were far more challenging [20] and the peers struggled much more to get acceptance. The peer supporters required substantial supervision and follow-up from their respective trainers and supervisors. Issues like no previous job experience, own health problems, emotional stress through fear and rejection, safety issues and HIV disclosure issues among study mothers were of far greater concern in the study than the intervention itself [21]. In other words, the South African peer supporters expressed a need for far deeper and more comprehensive understanding for themselves, combined with supervision and follow-up. With South Africa’s high HIV prevalence, it could be argued that a community intervention alone without intervening at facility-level was not enough to alter behaviour. However, we report findings from the HIV-negative and breastfeeding intending South African women in this paper so HIV as such cannot explain all the challenges this intervention had in South Africa. Furthermore, the counsellors were not recruited from the communities in the same way as in Burkina Faso and Uganda, thus, did not provide the same sense of community belonging. They were geographically from the respective cluster area, but recruited in a more formal manner through advertisements and interviews which is the regulated way to identify and appoint personnel.

The issues related to the country specific implementation of the intervention might explain some of the variation between the countries. However, this study suggests that large country variations in feeding were present from birth. For example, it is known that formula feeding had a high acceptance in South Africa [34], whereas this is not the case in Uganda and Burkina Faso and large areas of sub-Saharan Africa where it is considered unaffordable, unacceptable and unfeasible [35]. Women in Burkina Faso and Uganda might thus have been more motivated to breastfeed than in South Africa. Another country difference is that Burkina Faso has less access to and use of maternity facilities and lower general educational level, thus, this might explain why e.g. traditional practices illustrated by e.g. delaying and giving drops and syrups to the child had a high uptake there. This understanding was also supported from parallel research within the prevention-of-mother-to-child transmission of HIV (PMTCT) research [36]. Thus, timing of initiation of breastfeeding did not improve substantially in this trial, and the community-based intervention was not sufficient to get an effect on the timing of breastfeeding in Burkina Faso.

### Prelacteal feeds and drops or religious feeds

We presented the relationship between prelacteal feeds and drops or religious feeds. There was a strong relationship between these two practices in all three countries. However, smaller babies in Burkina Faso and South Africa got more prelacteal feeds that may indicate that mothers believed their babies needed this to a larger extent.

### Colostrum and initiation of breastfeeding

In contrast to the other two countries a behaviour change was seen from the intervention in Uganda related to colostrum and initiation of breastfeeding, though, relatively moderate. Mothers in the intervention group were more likely to give colostrum to their babies in comparison to control cluster mothers. This could serve as an indication for earlier behaviour change in Uganda compared to the other countries as mentioned [31], and that they perceived and recognised the recommendation “immediate” and “exclusive” in this setting. In line with our study, comprehensive and integrated counselling strategies have been described as very successful in changing behaviour and health outcomes, as was the case from a large trial in Haryana, India [37, 38]. Further, subjective intrinsic factors determining behaviour change are deeply embedded in cultural norms and traditions, thus understanding context of relevant barriers related to self-efficacy, beliefs and attitudes is key to meet and counsel the women in a meaningful way [38, 39]. Our study group addressed some of these factors in formative research identifying poverty and limited access to health systems facilities, water, sanitation and fear of stigma and rejection as key factors why not breastfeeding is considered a non-option in Burkina Faso and Uganda [33, 40]. In a way in these two countries, breastfeeding was already an established norm, and the trial had to address the question whether “breastfeeding was really enough.” The peer-intervention training package provided enough essential information on stimulation and production of breast milk to address this [31].

The usefulness of theory to inform and guide health behaviour interventions cannot be underestimated. While the intervention was not planned and implemented using a specific theory *a priori*, the intervention has been described [32] according to Kok and colleagues’ work [41] on Intervention Mapping: The theory describes a flow for implementation originating from proximal programmes through the development of strategies, programme plans, adaption and evaluation. This flow is implying certain consecutive tasks. In our case the initial step was the WHO course which was adapted by the research team and through local knowledge (research team in collaboration with the communities) moderated and operationalised. However, this process was not uni-linear, but involved substantial feedback options through interaction and pre-testing [19, 32, 42].

### Strengths and limitations

This analysis is based on self-reported, not observed, early infant feeding practices, hence there is the potential for information bias. However, the high number of women reporting prelacteal feeds in the intervention group (12%, 15% and 25% in Burkina Faso, Uganda and South Africa, respectively) might indicate that these questions were well understood and responded to. The PROMISE-EBF trial did not capture birth weight, however, we asked for birth weight at the 3-week interview. Using 3-week anthropometric information as a proxy for birth weight is a limitation because, our outcome of interest, early infant feeding, happened in real time before the measurements. This study does not allow us to investigate any relationship between early severe events such as neonatal death or hospitalisation due to a) sample size limitations; and b) limitation in study design resulting in healthy live children being enrolled in the follow-up where the early infant feeding questions were asked. However, studies on peri- and neonatal mortality exist from the cohort of pregnant women enrolled from both Burkina Faso and Uganda without reporting on early infant feeding practices, while indicating associations between severe outcomes (death) and place of delivery and parity in Uganda and Burkina Faso, respectively [43–45].

## Conclusion

The PROMISE-EBF trial showed that breastfeeding counselling by peers was associated with less prelacteal feeding in Burkina Faso and Uganda. More children received colostrum and started breastfeeding early in Uganda which means that in Uganda, mothers might have perceived the “exclusive” and “immediate” message to a large extent. There were substantial country variations, while no clear behaviour change was seen in South Africa. There is a need to understand safe and efficient ways to improve early infant feeding practices and how to create efficient and sustainable strategies for this. Public services and community health workers or peers would need to streamline messages to the pregnant women. This would demand regular health worker refresher training, supervision and remuneration: willingness and resources.

## Authors’ information

Ingunn Marie S Engebretsen and Victoria Nankabirwa share first authorship.

## Electronic supplementary material

Additional file 1: **Post-hoc power calculation.** (DOCX 15 KB)
