# Supplementary material for: Early infant feeding practices in three African countries: the PROMISE-EBF trial promoting exclusive breastfeeding by peer counsellors
Source: Int Breastfeed J. 2014 Nov 18;9:19. doi: 10.1186/1746-4358-9-19 (PMC4362641; doi:10.1186/1746-4358-9-19)
Supplement: Supplementary file 1 — Additional file 1: Post-hoc power calculation. (DOCX 15 KB) [file 13006_2014_192_MOESM1_ESM.docx]

# Additional file 1: Post-hoc power calculation

A post-hoc calculation of power for our main outcomes of interest (prelacteal feeds, initiation of breastfeeding before one hour (initiation < 1h), and expressing and discarding colostrum (no colostrum) is given below, generally the power was very high. Calculations were made according to [www.statisticalsolutions.net](http://www.statisticalsolutions.net) and the following given: A 2-tailed test, alpha of 0.05, the differences in mean, the varying number of measured children at 24 weeks (Table 1) gave the following results:

|  | **Burkina Faso** | | | **Uganda** | | | **South Africa** | | |
| --- | --- | --- | --- | --- | --- | --- | --- | --- | --- |
|  | Intervention | Control | **Power** | Intervention | Control | **Power** | Intervention | Control | **Power** |
| **Prelacteal feeds** | 41/372 (11.0) | 135/371 (36.4) | 1.00 | 52/387 (13.4) | 153/352 (43.5) | 1.00 | 145/480 (30.2) | 144/437 (33.0) | 1.00 |
| **Initiation < 1 h** | 14/392 (3.6) | 14/402 (3.5) | 0.78 | 219/396 (55.3) | 151/369 (40.9) | 1.00 | 252/535 (47.1) | 248/485 (51.1) | 1.00 |
| **No colostrum** | 30/371 (8.1) | 46/371 (12.4) | 1.00 | 10/382 (2.6) | 33/349 (9.5) | 1.00 | 83/478 (17.4) | 68/439 (15.5) | 1.00 |
